# From science to politics: COVID-19 information fatigue on YouTube

**DOI:** 10.1186/s12889-022-13151-7

**Published:** 2022-04-23

**Authors:** Chyun-Fung Shi, Matthew C. So, Sophie Stelmach, Arielle Earn, David J. D. Earn, Jonathan Dushoff

**Affiliations:** 1 Department of Biology, McMaster University, Hamilton, ON Canada; 2 Max Rady College of Medicine, University of Manitoba, Winnipeg, MB Canada; 3 Department of Mathematics and Statistics, McMaster University, Hamilton, ON Canada; 4 The Department of Ecology and Evolutionary Biology and the Department of Political Science, University of Toronto, ON Toronto, Canada; 5 Michael G. DeGroote Institute for Infectious Disease Research, McMaster University, Hamilton, ON Canada

**Keywords:** COVID-19, YouTube, Social media, Misinformation, Ealth communication, Pandemic

## Abstract

**Objective:**

The COVID-19 pandemic is the first pandemic where social media platforms relayed information on a large scale, enabling an “infodemic” of conflicting information which undermined the global response to the pandemic. Understanding how the information circulated and evolved on social media platforms is essential for planning future public health campaigns. This study investigated what types of themes about COVID-19 were most viewed on YouTube during the first 8 months of the pandemic, and how COVID-19 themes progressed over this period.

**Methods:**

We analyzed top-viewed YouTube COVID-19-related videos in English from December 1, 2019 to August 16, 2020 with an open inductive content analysis. We coded 536 videos associated with 1.1 billion views across the study period. East Asian countries were the first to report the virus, while most of the top-viewed videos in English were from the US. Videos from straight news outlets dominated the top-viewed videos throughout the outbreak, and public health authorities contributed the fewest. Although straight news was the dominant COVID-19 video source with various types of themes, its viewership per video was similar to that for entertainment news and YouTubers after March.

**Results:**

We found, first, that collective public attention to the COVID-19 pandemic on YouTube peaked around March 2020, before the outbreak peaked, and flattened afterwards despite a spike in worldwide cases. Second, more videos focused on prevention early on, but videos with political themes increased through time. Third, regarding prevention and control measures, masking received much less attention than lockdown and social distancing in the study period.

**Conclusion:**

Our study suggests that a transition of focus from science to politics on social media intensified the COVID-19 infodemic and may have weakened mitigation measures during the first waves of the COVID-19 pandemic. It is recommended that authorities should consider co-operating with reputable social media influencers to promote health campaigns and improve health literacy. In addition, given high levels of globalization of social platforms and polarization of users, tailoring communication towards different digital communities is likely to be essential.

**Supplementary Information:**

The online version contains supplementary material available at 10.1186/s12889-022-13151-7.

## Background

The coronavirus SARS-CoV-2 spread coronavirus disease (COVID-19) around the world in early 2020 [1], causing widespread panic [2, 3]. It brought an unprecedented global public health crisis that drew comparisons to the 1918 Spanish flu (e.g., [3, 4]), causing economic and social disruption and uncovering inequalities in health care [2, 3, 5]. COVID-19 was the first pandemic to occur at a time when social media platforms were used on a massive scale [6, 7], enabling an “infodemic” [8] of conflicting information which undermined the global response to COVID-19 and jeopardized measures to control the pandemic [7, 9]. Social media contributes to how the public views and responds to a health crisis [10–13] and affects policy responses and mitigation of epidemic spread [14, 15].

As the leading online social platform in the United States [16] and the second most popular social media site in the world next to Facebook [17], YouTube has been a major source of medical information during the COVID-19 pandemic [18, 19]. There have been studies of COVID-19-related content on YouTube, mostly focused on the early outbreak, and on specific topics (e.g., [20–24]). An early COVID-19 information study compared information in YouTube videos with U.S. CDC prevention guidelines; the authors found that fewer than one-third of the videos covered the key prevention behaviors listed by the CDC [23]. A similar study analyzing Spanish videos also concluded that information on basic measures to prevent SARS-CoV-2 spread was usually incomplete [25]. A study of Korean YouTube videos found that videos about COVID-19 from different sources varied significantly in terms of reliability, and that misleading videos tended to have more likes [26]. Similarly, in the UK, the YouTube videos with highly politicized health content received more public engagement than other types of videos [24]. Due to the public’s lack of trust in their decision making, government officials and politicians could not produce a shared sense of inclusion concerning protective guidelines against the COVID-19 outbreak [27].

While there have been investigations of themes, sources, and/or engagement on YouTube [24, 28] or other social media [20], the studies to date have been limited regarding long-term changes of a COVID-19 focus in social media, especially with respect to integrated analysis of themes and sources along the first wave of COVID-19. Therefore, in this study we focus broadly on which COVID-19-related themes on YouTube attracted the most collective public attention (measured by view counts) over a longer period of time, and how these themes developed over time and competed with each other for public attention. The spread of political and informational polarization across social media can have negative consequences for public health [29], including reductions in mask-wearing behavior [30] and increased vaccine hesitancy [31, 32]. The reason is that the public typically responds to a public health crisis based on perceptions mediated by media (both traditional and social) and by public speculation, which can interfere with governments’ ability to make decisions and guide behaviour according to scientific evidence [33, 34].

On social media, all users (e.g., laypersons (YouTubers), media outlets, and governments) can be both content generators and consumers, and they all compete for public attention to their messages (i.e., view counts). This competition can affect public awareness and the ability of health organizations to promote their presumably helpful messages [35, 36]. Here we aimed to understand what COVID-19 topics attracted most public attention, and how the salient topics changed through the course of the first eight months of the pandemic. This paper aims, first, to identify what types of themes related to COVID-19 were most viewed on YouTube? Second, how did COVID-19 themes evolve over the course of the pandemic? Third, how were themes presented by health authorities different from themes presented by other sources?

## Methods

### Data and search strategy

Data were collected on August 25, 2020, using the YouTube API v3 in Python. We found the top up-to-50 most viewed videos released each week from December 1, 2019 to August 16, 2020 by searching for the keywords “coronavirus,” “COVID” and “Wuhan.” In addition, “SARS” was used as a keyword before February 2020 to include videos about the SARS-like disease before it was known to be caused specifically by SARS-CoV-2. The start date was chosen such that it would slightly predate the pandemic, while the end date was chosen to be close to the date of collection, while ensuring that each video would have at least one week of time to accumulate views before the date of collection.

**Fig. 1:**
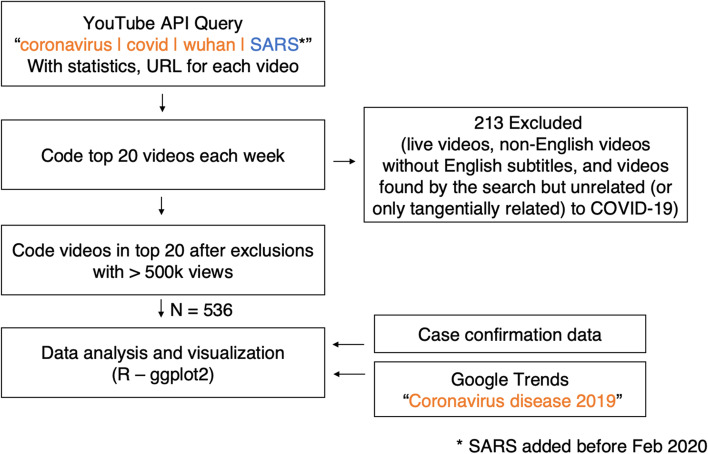
A summary of the data selection. 536 videos were selected for viewing and analysis. Videos were selected if they were in the top 20 of our original keyword search, or if they received over 1/2 million views and were in the top 20 after exclusions

Each week, videos were selected if they were identified (as previously described), as being in the top 20 most viewed videos of our original keyword search, or if they received over half a million views and were in the top 20 after exclusions. The 1/2 million views was arbitrarily decided as a practical cutoff point for a relatively popular video; we did not alter this cutoff based on the geographic origin of each video because most videos can accrue views from anywhere in the world. The following were excluded: live videos, non-English videos without English subtitles, and videos found by the search but unrelated (or only tangentially related) to COVID-19. Some of the early weeks had only a few qualifying videos. Google search interest for the topic ``Coronavirus disease 2019’’ from the weekly Google Trend data was collected from December 1, 2019 to August 16 for comparison with YouTube videos view interests. The search interest data of India, the United Kingdom, the United States and the world were selected for comparison (in Fig. 1) based on the the countries origin of the top viewed videos in English. Videos from India were mostly live news, and were not included in the analysis.

### Coding and statistics

We developed a coding book using an open inductive coding approach [37]. Instead of pre-determined coding frames, we reviewed and interpreted sample videos to generate concepts at the initial stage of developing a coding book, following the guidelines in previous research [19, 23, 38], and guidelines from the WHO [1] and the CDC [39]. We finalized the coding book after multiple rounds of practice coding and consultation among the coders; this process resulted in eleven themes in the coding book (Virus information; Statistics; Treatment; Prevention and action; Impacts on Non-Physical Health Related Issues (including anxiety, fears and other mental health content), Economic impact; Politics; International Cooperation, Human interest, Side Story, and Other), later grouped into 9 themes for analysis due to sample size (see Tables 1 and 2 in the supplement). Videos were coded for a main theme and an optional secondary theme; the analysis here is based on the main theme only.

There were two coders (AE and SS), both of whom had participated in the creation of the coding book, as well as having multiple rounds of coding practices. Consensus was achieved by discussion between coders and the first author (CS). Each video was assigned a code by one of the coders. Both coders also coded 5 percent of the videos assigned to the other to assess inter-coder reliability.

We evaluated the clarity [40] of observed patterns in view-count distributions across sources and themes using the Kruskal–Wallis test (kruskal.test in R). Ribbons were added to time series using the loess smoother in ggplot2 in R, with span values chosen manually to assist visualization (see online code and comments). They do not represent principled confidence intervals. Patterns of association between theme and source were evaluated with chisq.test in R, using the “simulate p values” option to avoid estimation problems.

## Results

Cohen’s Kappa statistic for inter-coder reliability was 0.76, which is considered acceptable for content analysis [41]. Overall, 536 videos were selected and coded over 37 weeks. Of the videos in the weekly top 20 of the original keyword search, 213 were rejected (e.g., live news reports from Indian news channels). Figure 1 summarizes the search methodology described previously.

Collectively, the 536 coded videos were viewed over 1.1 billion times in total, as of August 25, 2020. The earliest videos selected for coding under our criteria were from Singapore on January 3rd, 2020 (see “Chinese authorities working to identify virus behind pneumonia outbreak in Wuhan” in Table 3 in the supplement) and from Hong Kong on January 4th (see “Mystery illness outbreak in Wuhan, China” in Table 3 in the supplement). The most-viewed video coded was “The Coronavirus Explained & What You Should Do” (see Table 3 in the supplement) with over 26 million views, on May 19th by Kurzgesagt, a professional YouTube channel run by a German animation and design studio. Most of the top-viewed videos were posted by US-based sources.

East Asian countries were the first to report the virus, but the United States became the leading country by view counts afterwards. After Hong Kong and Singapore, source countries in January 2020 (see Table 3 in the supplement) included.

- Korea on the 10th of January “New type of coronavirus found for pneumonia outbreak in China: WHO” (video number 11 in Table 3),
- Thailand on the 13th “Thailand reports first case of Wuhan coronavirus outside China” (video number 12),
- UK on the 18th “Could this coronavirus be Disease X? Everything you need to know about the mystery virus in China” (video number 13),
- USA on the 19th “See where officials believe the coronavirus started” (video number 14),
- China on the 23rd “Coronavirus patient in Wuhan expected to leave hospital after ECMO support” (video number 15), and
- Canada on the 25th “Coronavirus Q&A: Separating fact from fiction” (video number 16).

**Fig. 2:**
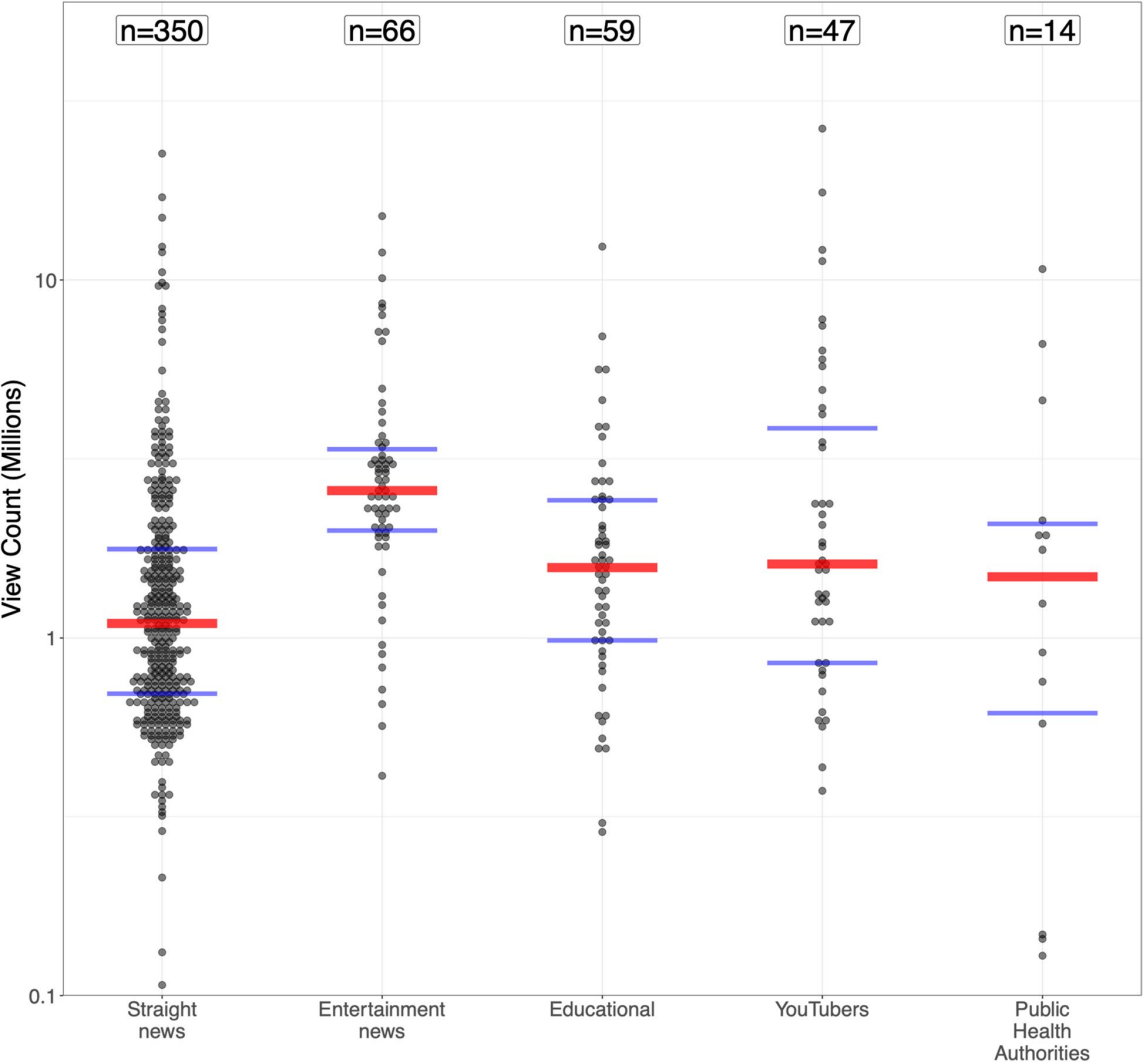
View counts per video, grouped by source, shown on a log scale. Each video is represented by a black dot. The red line shows the median, while blue lines signify the upper and lower quartiles. Sources are listed in decreasing order of coded videos. Some sources have videos that are not shown due to having less than 100,000 views: YouTubers (4.3% missing, minimum = 1,643), Educational (1.7% missing, minimum = 35,755), straight news (1.1% missing, minimum = 23,848)

Figure 2 shows videos broken down by video source (see Tables 4 in the supplement), in decreasing order of number of coded videos. Straight news (e.g., ABC news, CNN, Fox News) contributed the largest number of coded videos (65%), and the public health authorities (WHO and Governments combined) the least (3%). Entertainment news (e.g., Last week tonight with John Oliver) received the highest median views of 2.6 millions per coded video (see Table 5 in the supplement). The Kruskal–Wallis test yielded *p* < 0.001 for overall differences between medians, indicating an ability to see some clear differences among view counts for the different video sources.

**Fig. 3:**
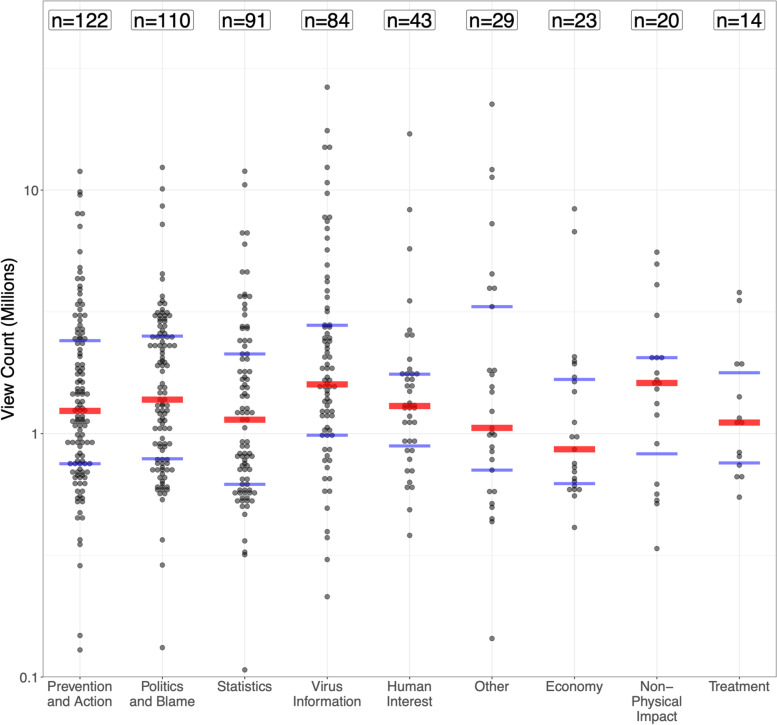
View counts per video, grouped by theme, shown on a log scale. Each video is represented by a black dot. The red line shows the median, while blue lines signify the upper and lower quartiles. Some themes have videos that are not shown due to having less than 100,000 views: virus information (6.0% missing, minimum = 1,961), human interest (2.3% missing, minimum = 1,643), statistics and prevention (1.1% missing, minimum = 23,848)

Figure 3 shows videos broken down by theme, in decreasing order of video counts. Virus Information and Non-Physical Impacts received the highest median views with about 1.6 million views per video vs. 0.9 million in Economy, the least viewed theme (see Table 2 in the supplement). Kruskal–Wallis *p* = 0.111 for overall differences between medians, indicating that the pattern of differences in view counts by themes is not clear.

**Fig. 4:**
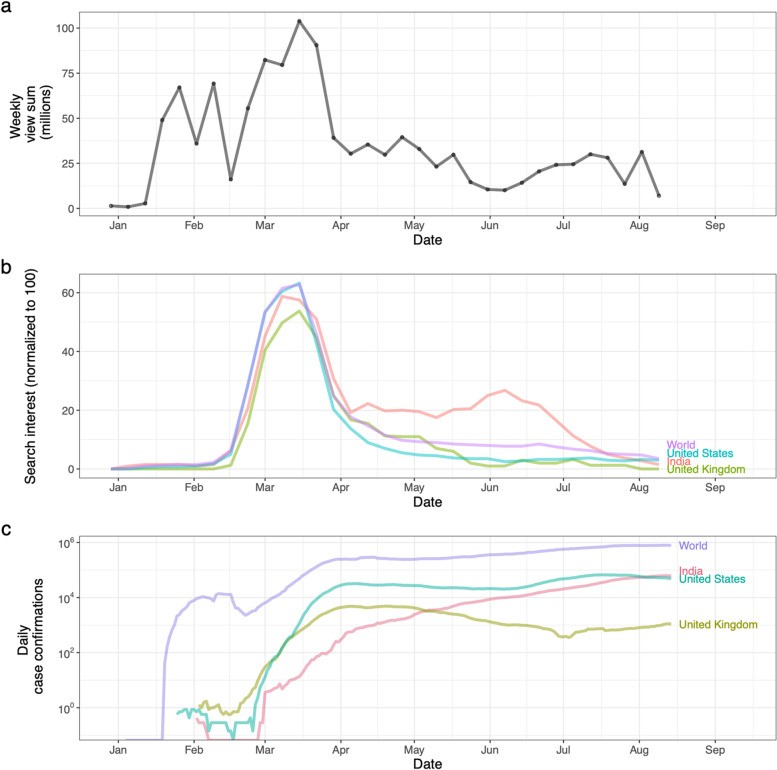
a) Total number of views of all coded videos per week. b) Weekly Google Trends normalized search interest for the topic Coronavirus disease 2019, smoothed with a 4-week rolling mean, from the Google Trends webpage. (Note that the week with the highest interest for each region would have a search interest of 100, but the peak height is reduced due to smoothing.) c) Daily COVID-19 incidence in major English-speaking nations, and the world as a whole (from ``Our World in Data’’ [42]), smoothed with a 7-day rolling mean

**Fig. 5:**
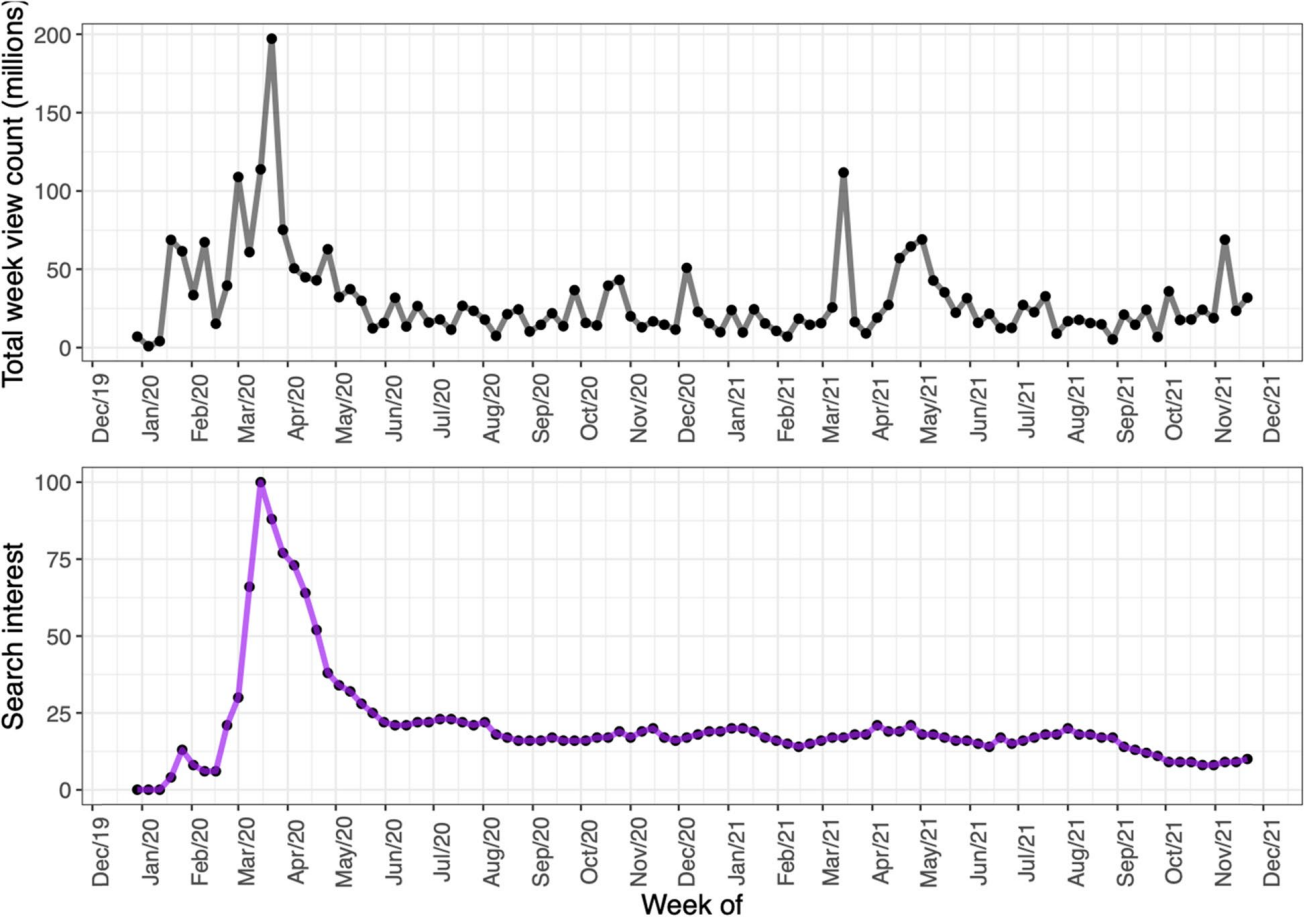
Top: The total number of views accumulated over the top 20 most viewed YouTube videos in each week, updated on December 7, 2021 using the YouTube API downloader code with the same keywords for this study. The view counts in this figure differ from panel a in Fig. 1 most likely due to videos having more time to accumulate views, as well as potentially some videos becoming unavailable. Bottom: The worldwide Google Trends ``search interest’’ for the topic Coronavirus disease 2019}, updated on December 13, 2021

Figure 4 shows the total number of views of the coded videos per week by publication date as of August 25, 2020. The total number of views for videos coded increased between January and March 2020, reaching a peak in Mid-March 2020 and declining afterwards (panel a). This pattern is somewhat similar to the Google Trends “search interest” for the topic Coronavirus disease 2019 (panel b); however, the search interest does not start rapidly increasing until mid-February 2020. YouTube views and Google search interest both peaked in mid-March and declined afterwards, even while COVID-19 incidence was still increasing (panel c). For comparison, we also researched YouTube views and Google Trends search interest after the research period. The overall patterns of a sharp increase in search interest in March–April 2020, followed by a decrease in collective attention, were continued for more than a year after the original study period. (See Fig. 5).

**Fig. 6:**
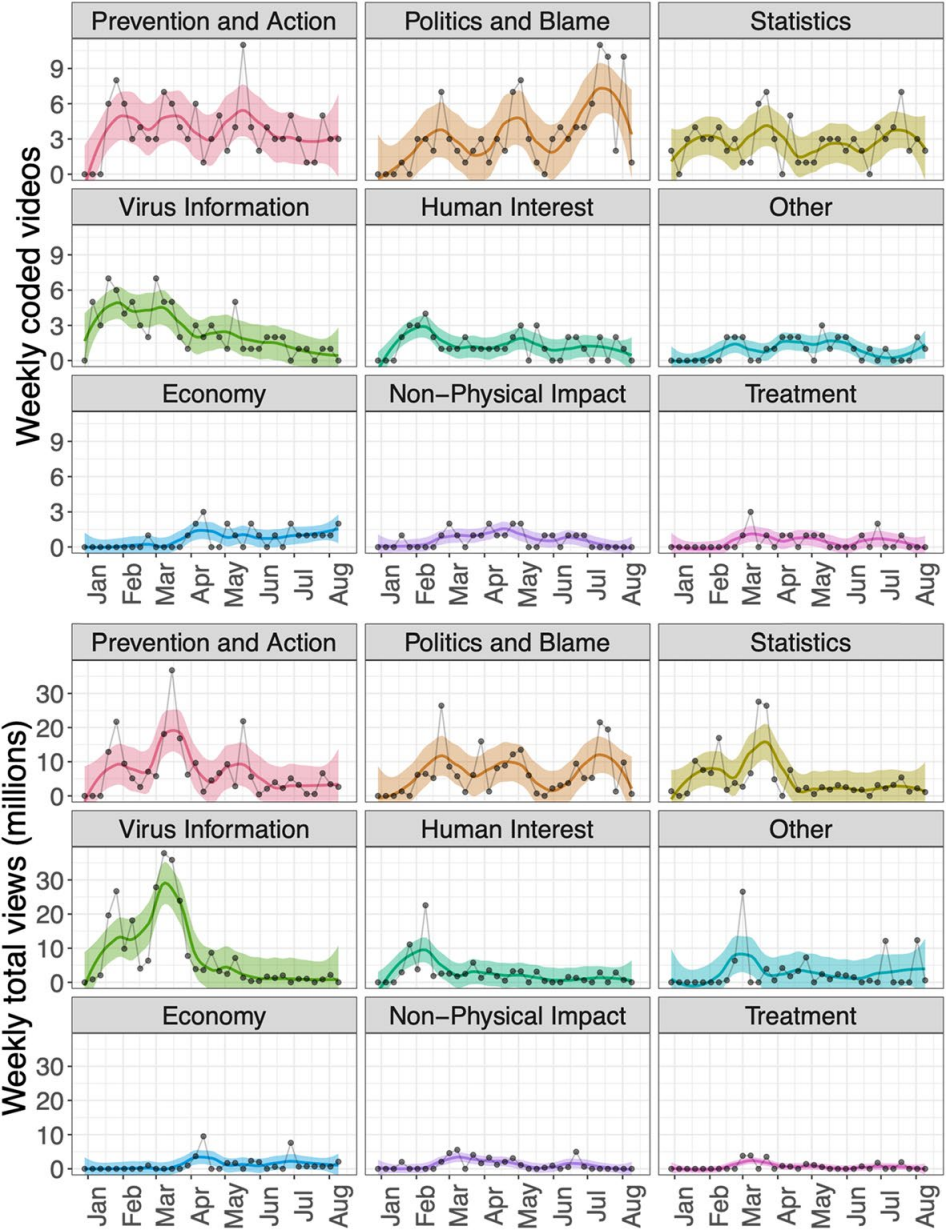
Smoothed patterns of numbers of videos published by theme (top) and corresponding weekly total views (bottom)

Figure 6 shows video views by theme over the outbreak. The three themes with the most top-viewed videos are (top panel): Prevention and Action, Politics and Blame, and Statistics. Videos about Virus Information, and Prevention and Action were the most popular themes published in March, resulting in a high overall view counts during this time (Fig. 4).

**Fig. 7:**
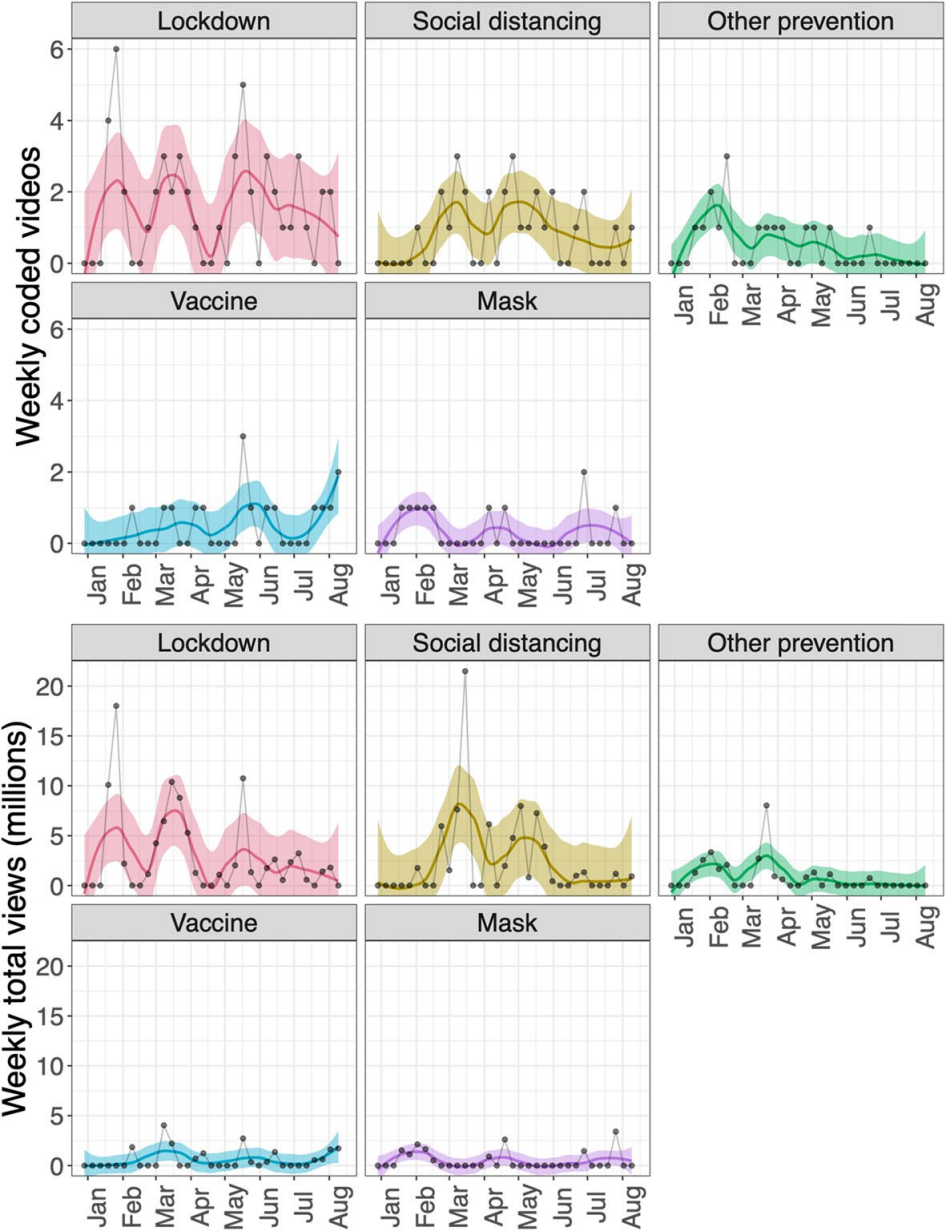
Smoothed patterns of numbers of videos published by prevention sub-theme (top) and corresponding weekly total views (bottom)

Figure 7 shows sub-themes coded under “prevention,” which included lockdown, social distancing, vaccine and mask. Overall, the largest numbers of videos were on topics related to lockdown, followed by social distancing; popular videos about vaccines increased in August (top panel). Videos about Lockdown and Social distancing also received the highest number of views (bottom panel). It is notable that Vaccines and Masks had both low weekly total view counts and few videos with enough views to be coded. It is also important to note that this study was conducted before the social media attention about vaccines when the first COVID-19 vaccines were approved for emergency use in early December 2020 [43, 44].

**Fig. 8:**
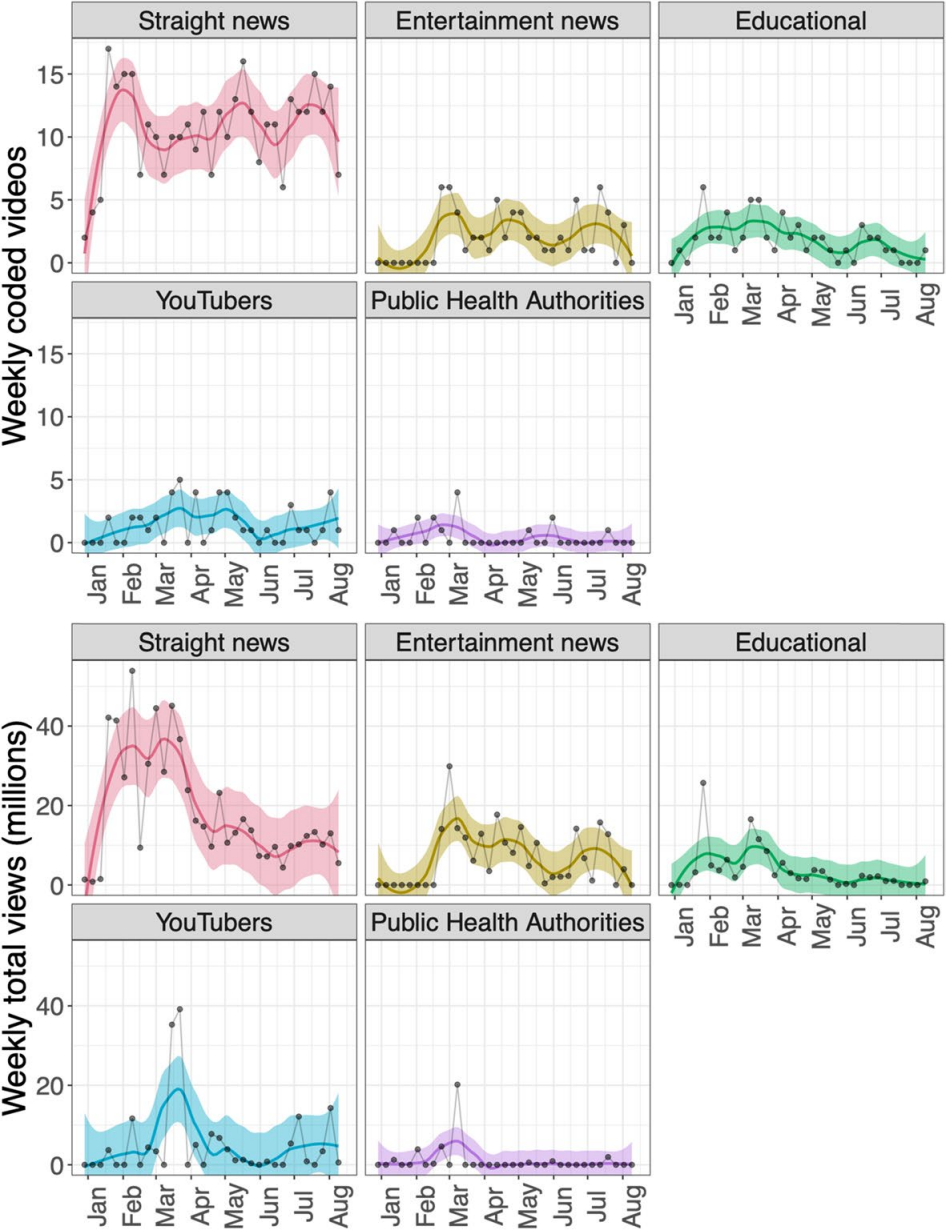
Smoothed patterns of numbers of videos published by source (top) and corresponding weekly total views (bottom)

Figure 8 shows the distribution of video sources over time: Videos published by straight news outlets dominated the top-viewed videos throughout the outbreak, and public health authorities contributed the fewest top videos (see the top panel). Although straight news was the dominant COVID-19 video source on YouTube, after March its viewership per video was similar to other outlets, such as entertainment news (e.g., Last Week Tonight with John Oliver) and YouTubers (e.g., Doctor Mike Hansen, and MedCram).

**Fig. 9:**
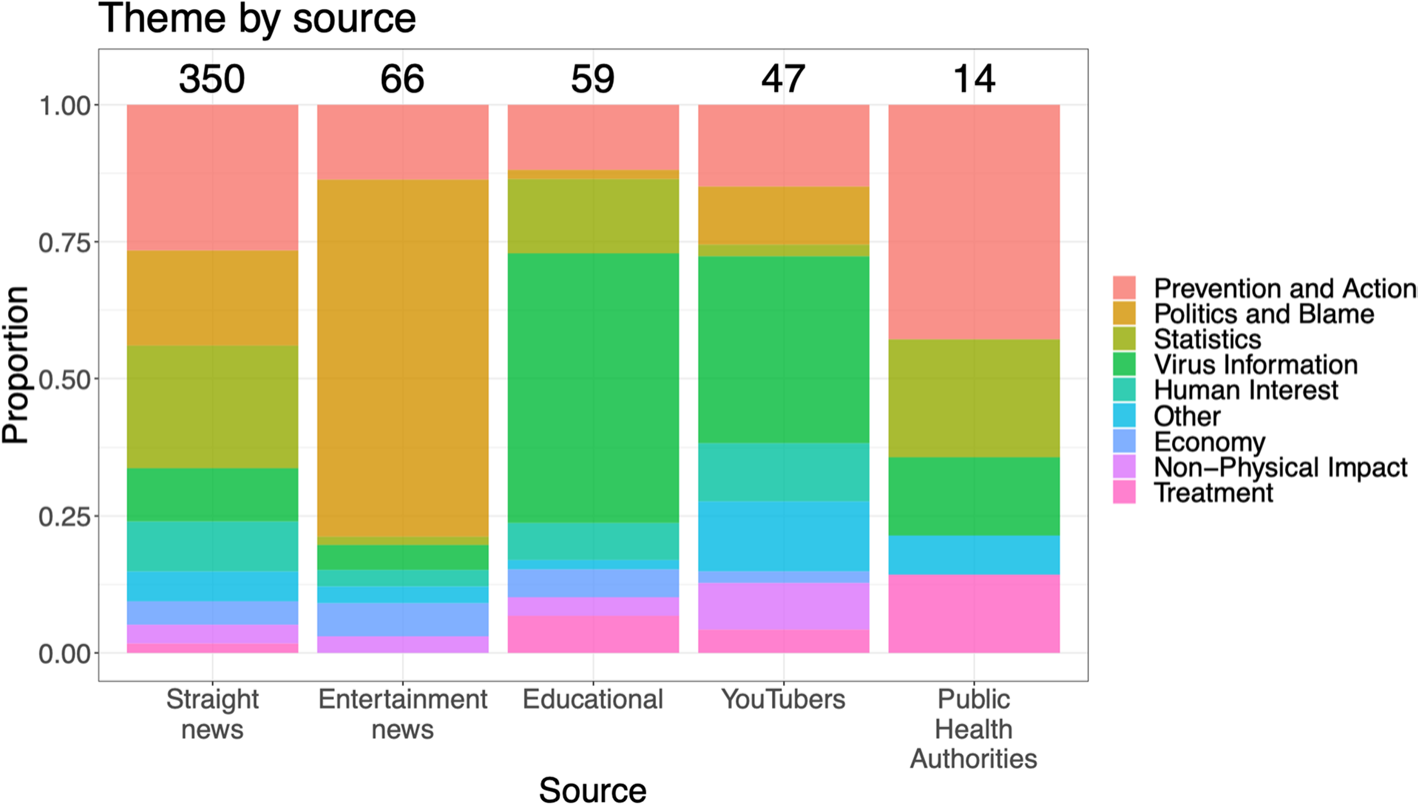
Distribution of video themes across sources. Sources are ordered according to number of coded videos. ${\chi }^{2}$  $p<0.001$, indicating a clear overall pattern of association between source types and theme types.

Figure 9 shows the degree to which the various COVID-19-related themes were featured according to source type. The themes that featured most varied among the different source types.

- Straight news provided the most even coverage of all of the themes, whereas Entertainment news focused more on politics and blame (e.g., “Coronavirus IV: Last Week Tonight with John Oliver” in Table 3 in the supplement),
- Educational channels (e.g., see Johns Hopkins University’s “Experts Brief Capitol Hill on Coronavirus” in Table 3 in the supplement) and YouTubers (e.g., MedCram’s “Coronavirus Symptoms, Diagnosis, Treatment” in Table 3 in the supplement) focused more on virus information.
- Less than 3% of the top-viewed videos were by public health authorities, the main sources being the WHO and the U.S. CDC. These videos mainly focused on prevention and updates on the COVID-19 outbreak. WHO had the greatest emphasis on Prevention and Action (e.g., “What can people do to protect themselves and others from getting the new coronavirus?” in Table 3 in the supplement), while the CDC’s videos were typically COVID-19 updates (e.g., “CDC Briefing Room: COVID-19 Update and Risks” in Table 3 in the supplement).

## Discussion

The main findings of this study were that collective attention to COVID-19 on YouTube peaked around March 2020 and flattened out until at least mid-August 2020. Early videos focused on prevention, but politics and blame became a more salient theme over time. Politics and blame received the most public attention, amassing the largest number of collective views as well as having high median comment and like counts.

In this study, we analyzed 536 highly viewed COVID-19-related videos posted on YouTube from December 1, 2019 to August 16, 2020. The earliest videos were from Singapore and Hong Kong, and reported a then-mysterious virus and the Chinese authorities’ claim of no evidence of human-to-human transmission. The first top-viewed video posted by WHO was “Q&A about coronavirus” (see Table 3 in the supplement) uploaded on January 16, after the organization announced that China had identified a novel coronavirus on January 7, 2020 [1]. Straight news outlets posted the majority of the top-viewed videos, while public-health authorities posted the smallest number of top-viewed videos. The themes of Prevention and Politics received the largest number of collective views, while Treatment received the least (Fig. 3).

Based on our findings, we propose that, first, collective public attention to the COVID-19 pandemic on social media peaked early and flattened afterwards (see Fig. 4), likely due to information fatigue [45, 46]. Second, there was a clear shift of COVID-19 themes from prevention early on (22.8% of total videos coded) to politics and blame later (20.5% of total videos coded, which is clearly different from the early 22.8% prevention, based on a two-sided test of equal proportions using prop.test in R, *p* < 0.001). 27/225 (12.0%) videos published before April 8, 2020 had themes of politics and blame, compared to 83/331 (26.7%) videos published on or after April 8, 2020 (see Fig. 6). Videos oriented to politics and blame also received more reactions from the viewers as measured by comments and likes/dislikes (see Table 6 in supplementary material). That politics and blame became the most salient theme among the top-viewed videos after April could be due to the politicization of COVID-19, influenced especially by the upcoming presidential election in the U.S. during that time [29, 47] (see Fig. 6). Third, regarding prevention and control measures, masking received much less attention than lockdown and social distancing in our study period (Fig. 7).

Videos published before April 2020 received the most attention, and the majority of the videos during that time focused on basic information about the virus, the disease, and prevention guidelines (see Figs. 4 and 6). Although we are not able to confirm when views occurred, this pattern, together with similar patterns in Google search trends (see Fig. 4 and other studies [48–50]), likely point to attention fatigue. Despite spikes of COVID-19 cases, it appears that collective attention to the outbreak (at least on YouTube and Google searches) decreased after March 2020. This decline could indicate that the public was overwhelmed by COVID-19-related information, that they felt that they understood enough about the disease, or that they simply became less interested. Attention fatigue could have impacted health-related behaviors, increasing behaviors that went against regulations and worsened epidemic severity [46]. It is also plausible that the initial panic that overwhelmed many as the virus emerged subsided as more information became available [6]; and what followed was a shift to interest in political themes. Highly politicized messages tend to receive more engagement on YouTube [24], and distrust and competition among information sources can reduce dissemination of presumably helpful health information [27, 35], as an “infodemic” of conflicting information means that valuable information cannot be communicated clearly [9].

The public’s perception of crises can be shaped by media content [13, 35, 51, 52], as can behavioral responses [10, 13]. Fatigue might contribute to behavior changes [45, 46] (e.g., becoming less alert and paying less attention to distancing measures) and influence epidemic dynamics, especially when messages about controlling spread become less clear and more political [29]. When public attention switches to more controversial topics (e.g., political blaming games) and away from public health information (e.g., effectiveness of universal masking and non-physical COVID-19 impacts) during a public health crisis, what is missed or misread can be consequential.

For example, masking was not a major theme in top-viewed videos (Fig. 7) in the study time period. While search interest in how to make coronavirus masks spiked in May 2020 [50], universal masking in the United States was not proposed until the CDC called on Americans to wear masks to prevent the SARS-CoV-2 spread on July 14, 2020 [53, 54]. From “Can masks protect against the new coronavirus infection?” (in Table 3 in the supplements) on February 5, 2020, “Trump Ignored Coronavirus Warnings; Pence Refused to Wear a Mask: A Closer Look” (in Table 3 in the supplements) on April 26 and “Wear a mask. Help slow the spread of Covid-19” (in Table 3 in the supplements) on July 26, the psychological and public-health effects of mask wearing remained culturally and politically controversial as observed elsewhere [30], despite the spike of infected cases in the United States and worldwide (see panel c in Fig. 4). The politicization of masking, in addition to a public attention fatigue, served to undermine the control of SARS-CoV-2 transmission. Furthermore, given that the use of social media as a source of COVID-19 information is associated with fewer COVID-19 health-protective behaviors [21],it is possible that the misuse of social media sites could have increased COVID-19 cases and deaths (for example, vaccine hesitancy [55].

Politicization could extend also to vaccination and vaccine hesitancy due to ethno-cultural, religious, or political beliefs [55], as observed internationally [31], and in the UK and USA in particular [32]. Although there were few top-viewed videos about SARS-CoV-2 vaccines compared to other prevention methods, the number of vaccine-related videos increased while others decreased in August (Fig. 7). The early videos about vaccination tended to focus on the timeline of availability with the assumption that everyone wanted to be vaccinated, but the later vaccination discussion became more skeptical (e.g., “The risky way to speed up a coronavirus vaccine” in Table 3 in the supplement), and more political (e.g., “Half of Britons would not get a coronavirus vaccination” in Table 3 in the supplement). The social media discussion regarding vaccines (and vaccine hesitancy) may have increased after the study period, due to the development and approval of numerous vaccines in late 2020 [56]. Social media could have enhanced the politicization of vaccination and other public health measures, therefore reducing their effectiveness as vaccine hesitancy was likely associated with political ideology [57].

A shift from science to politics at an early stage could represent an important missed opportunity to disseminate useful information about prevention, as suggested in a study of YouTube videos very early in the epidemic [23]. This shift to politics contributed to the infodemic, and may have weakened mitigation measures during the first waves of the COVID-19 pandemic as government decisions are expected to be based on science [33, 34]. While political polarization may lead to an increase in the uptake of inaccurate information from individuals’ self-selected polarized news sources or echo chambers [58], the subsequent reduction in media and public attention to COVID-19 may also have made it more difficult for public health authorities to disseminate key information. We hope that our findings can contribute to raising awareness of the importance of science communication in combatting polarization and the spread of misinformation.

An important study limitation is that we were not able to access the view dates of videos, only publication dates. Thus, our knowledge of when viewing patterns changed is limited, and our comparisons between older and newer videos are potentially biased, since the former had more time to accumulate views. However, we note that overall patterns in our YouTube viewership are consistent with those found in Google Trends – in particular, the pattern of a mid-March spike in attention with a decline over the subsequent months. We recognized that mental health related issues should have been a theme apart from Impacts on Non-Physical Health Related Issues since mental health was strongly related to the pandemic [2, 59] and a detailed analysis should be considered in similar studies in the future. Another limitation is that, since we could not determine the viewers’ ethnic, cultural and political backgrounds, controlling for or comparing based on these factors was not possible. Consequently, our inferences concerning the patterns of themes and collective attention should be generalized cautiously.

## Conclusion

In summary, this study shows that collective media attention on YouTube related to the COVID-19 pandemic peaked in March 2020 and plateaued until the end of the study period, despite high worldwide COVID-19 incidence during the plateau period. Furthermore, the most salient theme in COVID-19 related videos quickly transitioned from prevention to politics, representing a politicization of COVID-19. Our findings have several implications. First, retaining public attention and convincing people to maintain precautions over time in the face of likely collective attention fatigue is a challenge that public health authorities should keep in mind. Authorities should consider co-operating with YouTubers to promote health campaigns. Second, given high levels of globalization and polarization, tailoring information towards different digital communities is likely to be very important. Third, immediate response to an emerging infodemic, and conspiracy theories before they go viral, will likely be crucial in future outbreaks; health authorities should be developing strategies now for how to respond quickly and effectively to combat misinformation and improve health literacy in the future. Future research that elucidates the themes and sources represented in COVID-19-related videos after August 2020 would be valuable, especially considering the development and subsequent misinformation regarding vaccines in 2020–2021.

## Supplementary Information

**Additional file 1:** **Table 1.** Coding themes. The theme usually appears right at the beginning of a video and on the headline and continues through the content. A theme is considered salient (coded as the main theme of a given video) when a video devotes more time to that theme than to others.

**Additional file 2:** **Table2.** Codedvideocountsbytheme. Thenumber(n)ofvideoswitheachtheme(percentage of total videos). The median (interquartile range) of views, likes, dislikes, and comments for videos with each theme. M denotes millions, while k denotes thousands.

**Additional file 3:** **Table 3.** Examples of Videos Viewed Meeting the Criteria for Inclusion from January to August 2020.

**Additional file 4:** **Table 4.** All types of sources identified and coded.

**Additional file 5:** **Table 5.** Coded video counts by source. The number (n) of videos with each source type (percentage of total videos). The median (interquartile range) of views, likes, dislikes, and comments for videos published by each source type. M denotes millions, while k denotes thousands.

**Additional file 6:** **Table 6.** Aggregate characteristics of all coded videos.

## Data Availability

The datasets analysed during the current study will be publicly available on our github repo when the paper is published.
