# Supplementary material for: From science to politics: COVID-19 information fatigue on YouTube
Source: BMC Public Health. 2022 Apr 23;22:816. doi: 10.1186/s12889-022-13151-7 (PMC9034744; doi:10.1186/s12889-022-13151-7)
Supplement: Supplementary file 1 — Additional file 1: Table 1. Coding themes. The theme usually appears right at the beginning of a video and on the headline and continues through the content. A theme is considered salient (coded as the main theme of a given video) when a video devotes more time to that theme than to others. [file 12889_2022_13151_MOESM1_ESM.pdf]

Table 1: Coding themes. The theme usually appears right at the beginning of a video and on the headline and continues through the content. A theme is considered salient (coded as the main theme of a given video) when a video devotes more time to that theme than to others.

**Theme**	**Explanation**
Virus Information	*The characteristics, natural history, symptoms, severity of disease, transmission, and epidemiological explanations of the coronavirus.*
Statistics, and Modeling	*The course of the pandemics, case, deaths, and hospitalization rates, supply of medical resources, and modelling predictions.*
Treatment	*Providing, evaluating, discussing, and suggesting methods of treatment for coronavirus patients, including information about what to do if suspicious of infection.*
Prevention and Action	*Discussion of prevention and control measures, whether as a policy or an option. Subcodes include: Lockdown and Reopening, Mask, Vaccine, Social distancing, Testing, contact tracing and sanitation, and Other.*
Impacts on Non-Physical Health Related Issues	*Broader scale of social impacts of the coronavirus, including anxiety, fears and other mental health issues, socialization, daily basic resources, and personal worries.*
Impacts on Economy	*Effects on business and on economic growth, relief packages, unemployment, stock performance, etc.*
Politics and Blame	*Discussion around the politics of coronavirus, often to do with who is to blame (At fault) for causing the virus, and/or prevention/action measures or lack thereof.*
Other: International Cooperation	*Cooperation and coordination by WHO and among nations, and humanitarian support.*
Human Interest	*Personal/community stories related to people's experiences during the pandemic. On a personal/individual/community level, rather than focusing on larger systemic issues.*
Other: Side Story	*For videos that mention COVID- briefly but not a main subject – only as a marginal point.*
Other	*None of the above.*
