# Supplementary material for: From science to politics: COVID-19 information fatigue on YouTube
Source: BMC Public Health. 2022 Apr 23;22:816. doi: 10.1186/s12889-022-13151-7 (PMC9034744; doi:10.1186/s12889-022-13151-7)
Supplement: Supplementary file 2 — Additional file 2: Table2. Codedvideocountsbytheme. Thenumber(n)ofvideoswitheachtheme(percentage of total videos). The median (interquartile range) of views, likes, dislikes, and comments for videos with each theme. M denotes millions, while k denotes thousands. [file 12889_2022_13151_MOESM2_ESM.pdf]

Table 2: Coded video counts by theme. The number (n) of videos with each theme (percentage of total videos). The median (interquartile range) of views, likes, dislikes, and comments for videos with each theme. M denotes millions, while k denotes thousands.

Theme	n (%)	Views (M)	Likes (k)	Dislikes (k)	Comments (k)
Prevention and Action	122 (22.8)	1.2 (1.7)	8.7 (19.9)	1.1 (1.9)	3.6 (5.1)
Politics and Blame	110 (20.5)	1.4 (1.7)	20.6 (31.0)	1.7 (1.4)	6.8 (4.9)
Statistics and Modeling	91 (17.0)	1.1 (1.5)	8.3 (16.0)	1.7 (2.2)	5.3 (5.8)
Virus information	84 (15.7)	1.6 (1.8)	18.8 (43.2)	1.2 (2.0)	3.6 (7.0)
Human Interest	43 (8.0)	1.3 (0.9)	19.0 (18.8)	1.0 (1.2)	3.4 (3.6)
Other	29 (5.4)	1.1 (2.6)	15.2 (23.5)	1.7 (3.8)	2.7 (3.8)
Economy	23 (4.3)	0.9 (1.0)	11.9 (19.0)	1.1 (2.5)	2.4 (4.9)
Non-Physical Impact	20 (3.7)	1.6 (1.2)	14.4 (30.9)	1.1 (0.9)	2.3 (6.3)
Treatment	14 (2.6)	1.1 (1.0)	10.7 (10.5)	0.8 (0.8)	2.7 (2.5)
All videos	536 (100)	1.3 (1.6)	12.7 (26.4)	1.3 (2.0)	4.5 (6.2)
