# Supplementary material for: From science to politics: COVID-19 information fatigue on YouTube
Source: BMC Public Health. 2022 Apr 23;22:816. doi: 10.1186/s12889-022-13151-7 (PMC9034744; doi:10.1186/s12889-022-13151-7)
Supplement: Supplementary file 3 — Additional file 3: Table 3. Examples of Videos Viewed Meeting the Criteria for Inclusion from January to August 2020. [file 12889_2022_13151_MOESM3_ESM.pdf]

Table 3: Examples of Videos Viewed Meeting the Criteria for Inclusion from January to August 2020.

Number	Title	Country
1	Chinese authorities working to identify virus behind pneumonia outbreak in Wuhan	Singapore
2	Mystery illness outbreak in Wuhan, China	Hong Kong
3	The Coronavirus Explained & What You Should Do	Germany
4	Ingraham: The real COVID record	USA
5	Coronavirus IV: Last Week Tonight with John Oliver (HBO)	United Kingdom
6	Johns Hopkins Experts Brief Capitol Hill on Coronavirus (COVID-19)	USA
7	Coronavirus Symptoms, Diagnosis, Treatment, & Vaccine Status	USA
8	What can people do to protect themselves and others from getting the new coronavirus?	N/A
9	CDC Briefing Room: COVID-19 Update and Risks	USA
10	WHO: Coronavirus - questions and answers (Q&A)	N/A
11	New type of coronavirus found for pneumonia outbreak in China: WHO	Korea
12	Thailand reports first case of Wuhan coronavirus outside China	Thailand
13	Could this coronavirus be Disease X?	United Kingdom
14	See where officials believe the coronavirus started	USA
15	Coronavirus patient in Wuhan expected to leave hospital after ECMO support	China
16	Coronavirus Q&A | Separating fact from fiction	Canada
17	Can masks protect against the new coronavirus infection?	N/A
18	Trump Ignored Coronavirus Warnings; Pence Refused to Wear a Mask: A Closer Look	USA
19	Wear a mask. Help slow the spread of Covid-19.	USA
20	The risky way to speed up a coronavirus vaccine	USA
21	COVID-19: Half of Britons would not get a coronavirus vaccination	United Kingdom
