# Supplementary material for: From science to politics: COVID-19 information fatigue on YouTube
Source: BMC Public Health. 2022 Apr 23;22:816. doi: 10.1186/s12889-022-13151-7 (PMC9034744; doi:10.1186/s12889-022-13151-7)
Supplement: Supplementary file 4 — Additional file 4: Table 4. All types of sources identified and coded. [file 12889_2022_13151_MOESM4_ESM.pdf]

Table 4: All types of sources identified and coded.

**Source Type**	**Explanation**
Straight News Outlet	Conventional/mainstream news media including newspapers, TV network, online news website. Outlet tends to produce journalistic-style videos, news broadcasts and interviews.
Entertainment News	Outlet reports news from an entertainment-based program; content made professionally, but not journalistic in style.
Public Health Authorities: Governments	Outlet provides news from governmental bodies and government agencies like regional health departments, (e.g., PHAC, CDC)
Public Health Authorities: WHO	Outlet for WHO news conferences, or presentations by WHO officials, or researchers on behalf of WHO.
Educational Groups	Outlet creates content by educational groups, academic institutions or hospitals; a verified non-governmental organization/ charity that is providing information about the coronavirus.
Youtubers: Professional	Individual or group with medical credentials.
Youtubers: Layperson	Individual or group with no medical/public health credentials or medical/public health affiliation.
