# Supplementary material for: From science to politics: COVID-19 information fatigue on YouTube
Source: BMC Public Health. 2022 Apr 23;22:816. doi: 10.1186/s12889-022-13151-7 (PMC9034744; doi:10.1186/s12889-022-13151-7)
Supplement: Supplementary file 5 — Additional file 5: Table 5. Coded video counts by source. The number (n) of videos with each source type (percentage of total videos). The median (interquartile range) of views, likes, dislikes, and comments for videos published by each source type. M denotes millions, while k denotes thousands. [file 12889_2022_13151_MOESM5_ESM.pdf]

Table 5: Coded video counts by source. The number (n) of videos with each source type (percentage of total videos). The median (interquartile range) of views, likes, dislikes, and comments for videos published by each source type. M denotes millions, while k denotes thousands.

Source	n (%)	Views (M)	Likes (k)	Dislikes (k)	Comments (k)
Straight news	350 (65.3)	1.1 (1.1)	8.9 (12.1)	1.3 (1.9)	4.4 (5.7)
Entertainment news	66 (12.3)	2.6 (1.4)	39.9 (31.2)	1.8 (1.7)	6.4 (5.2)
Educational	59 (11.0)	1.6 (1.4)	27.6 (49.7)	1.1 (1.6)	3.4 (6.3)
YouTubers	47 (8.8)	1.6 (3.0)	34.9 (52.2)	1.3 (3.0)	4.1 (7.3)
Public Health Authorities	14 (2.6)	1.5 (1.5)	3.3 (10.0)	0.8 (1.9)	0.3 (1.1)
All videos	536 (100)	1.3 (1.6)	12.7 (26.4)	1.3 (2.0)	4.5 (6.2)
