# Supplementary material for: From science to politics: COVID-19 information fatigue on YouTube
Source: BMC Public Health. 2022 Apr 23;22:816. doi: 10.1186/s12889-022-13151-7 (PMC9034744; doi:10.1186/s12889-022-13151-7)
Supplement: Supplementary file 6 — Additional file 6: Table 6. Aggregate characteristics of all coded videos. [file 12889_2022_13151_MOESM6_ESM.pdf]

Table 6: Aggregate characteristics of all coded videos.

	Mean (SD)	Median (IQR)
Views	2,131,818 (2,737,340)	1,288,288 (1,640,895)
Likes	32,039 (63,288)	12,705 (26,380)
Dislikes	2,686 (7,759)	1,326 (1,957)
Comments	6,360 (7,251)	4,504 (6,156)
Length (seconds)	639 (1,921)	430 (528)
Days	122 (62)	125 (105)
VPI	19,206 (27,296)	11,428 (13,640)
